# The Symptoms of Aneurism

**Published:** 1895-09-21

**Authors:** A. Pearce Gould

**Affiliations:** Senior Assistant Surgeon to the Middlesex Hospital


### The Symptoms of Aneurism.

By A. Pearce Gould, F.R.C.S., Senior Assistant Surgeon to the Middlesex Hospital.

The first and most important symptom of an aneurism is the presence of a tumour over and fixed to an artery. The recognition of a tumour is the first essential step in the diagnosis, and must never be omitted. It is necessary to lay stress upon this point, because inattention to it is the most frequent cause of mistakes in diagnosis. It is more common for an aneurism to be diagnosed when none is present than for the converse error to be made, the recognition of an abnormal pulsation being often held sufficient to indicate the presence of an aneurism. For example, an undue pulsation of the abdominal aorta felt at the epigastrium has often been mistaken for an aortic aneurism, especially when it is combined with pain in the back and symptoms of dyspepsia, which have been attributed to the pressure of the supposed aneurism upon the spine and the stomach. This error can always be avoided if it is remembered that until the observer has satisfied himself of the presence of a distinct tumour he is not in a position to diagnose an aneurism whatever other symptoms may be present. In the neck, too, tortuous or displaced arteries and pulsating veins may be mistaken for aneurisms if the same initial precaution be not taken. In the thorax and the cranium the verification of the presence of a tumour often depends upon other symptoms than those revealed by palpation, such as dulness on percussion and evidence of pressure upon the gullet or air passages, vessels or nerves. The tumours formed by an aneurism vary greatly in size, in shape, and in consistence, and also in its rapidity of formation and rate of progress. As a rule they are more or less globular in outline, and tense, and they sometimes have firm solid-feeling parts. The tumour, as already mentioned, is inseparable from the artery from which it springs; the converse of this symptom affords an easy way of distinguishing many tumours lying over and receiving pulsation from large arteries, such as enlarged glands in the neck or ham.

The second great group of symptoms of aneurism are those which result from the communication of the tumour with the lumen of the artery. If we remember the definition of an aneurism, as a "blood tumour communicating with an artery," we shall see that the symptoms of this communication form the second great pillar on which the diagnosis of aneurism rests. The more definite these evidences of communication are, the more valuable for diagnostic purposes. We will therefore take them in the order of value, and not in the order usually given, which is that of prominence or striking peculiarity. The most unequivocal of these signs of communication of the tumour with an artery is the shrinking of the tumour when the artery is compressed on the cardiac side, the still further yielding of the tumour under gentle pressure, and then its rapid filling out again with two or three throbs as soon as the pressure is taken off the artery. Next in value comes the expansile pulsation of the tumour; mere pulsation merely signifies proximity to an artery, but an actual expansion of the tumour with each beat of the heart results from the flow of blood from the artery into the aneurism. It is, therefore, very important to recognise this character of expansion of the tumour, and to distinguish it from mere displacement of the tumour such as may be caused in a movable tumour over a large artery. This sign is sometimes spoken of as "lateral pulsation," from the fact that the pulsation is felt at the sides of the tumour as well as over the summit, but it is better to use the term expansile pulsation because it emphasises the essential feature of the symptom—the filling out of the aneurism with each heart-beat.

Of much less diagnostic value is the bruit that is usually audible over an aneurism; when most characteristic it is systolic in time, blowing in character, and equally audible all over the tumour. It is never modified in character or intensity by the direction or degree of pressure of the end of the stethoscope, and this fact is of real importance, for it serves to distinguish the aneurismal bruit produced in the aneurism by the flow of blood into the sac, from the bruit caused by narrowing of an artery from the pressure of a tumour adjacent to it. The bruit, however, may be diastolic as well as systolic, and harsh, rasping, buzzing, or musical in character; again, in some cases, it is absent altogether. The thrill which is often, but not always, felt over an aneurism, is of still less diagnostic value; it is caused by the rush of blood from the artery into the sac, and sometimes probably, by the flow of blood over coagula within the sac.

The remaining symptoms of aneurism should be also arranged into two groups: 1, Those resulting from the interference with the circulation beyond the aneurism; and 2, those resulting from the pressure of the aneurismal tumour upon the surrounding structures—"pressure signs," as they are usually called. In the first group we have pallor and wasting of the parts beyond the aneurism from diminished blood-supply, a smaller and feebler pulse, and on comparing the pulse on the two sides of the body, that in the arteries below the aneurism is found to be a little delayed in time. On taking a syphygmographic tracing the percussion stroke may be found entirely lost on the side of the aneurism, making the tracing lower and more rounded at the top than on the other side. Associated with the comparatively empty state of the arteries below the aneurism there is often compensatory hypertrophy of the left ventricle of the heart. In many cases this is more dependent upon the general arterial disease than upon its local development into an aneurism.

The pressure signs of aneurism form a very important group of clinical phenomena, although in most cases they are of only secondary importance from a diagnostic point of view. The prominence of the pressure signs of an aneurism compared with those of other tumours arises from two circumstances. In the first place from the position of the main arteries of the body in close proximity to veins, nerves, and mucous canals, it happens that any swelling of an artery is so placed as of necessity to compress important structures. But, again, the pressure exerted by an aneurism is often far in excess of that exerted by any other form of tumour. In accordance with a well-known hydrostatic law that is exemplified in the Bramah press, the pressure of the sac of an aneurism containing only liquid blood is as many times greater than the pressure of the blood in the artery as the area of the sac is a multiple of the area of the artery. These pressure-signs are œdema, lividity, and venous distension from compression of veins, "neuralgic" pains and paralysis from compression of nerves, "obstruction" from compression of mucous canals, and local pain. This local pain is partly the result of pressure upon the terminations of nerves in the tissue around the sac, partly the result of the inflammation the pressure of the aneurism excites, and in some cases is due to the erosion of bone caused by the pressure of the aneurism. The pressure signs of an aneurism vary not only in intensity, but also in nature, with the position, size, and rapidity of growth of the tumour.

